# Evaluating the role of autistic traits and sensory sensitivity in eating disorders and autistic-like eating behaviours

**DOI:** 10.1192/j.eurpsy.2024.415

**Published:** 2024-08-27

**Authors:** G. Ingrosso, V. Nistico’, F. Lombardi, B. Morlacchi, A. C. Cigognini, R. Faggioli, A. Mottaran, M. Tramontano, L. Ranzini, C. A. Redaelli, S. Anselmetti, S. Bertelli, O. Gambini, B. Demartini

**Affiliations:** ^1^Health Science Department, University of Milan; ^2^Department of Psychology, University of Milan Bicocca; ^3^ Aldo Ravelli Research Center for Neurotechnology and Experimental Brain Therapeutics, University of Milan; ^4^UO Psichiatria 51 e 52, ASST Santi Paolo e Carlo, Presidio San Paolo; ^5^NutriMente Onlus, Milan, Italy

## Abstract

**Introduction:**

In recent decades, there has been extensive research on the association between Autism Spectrum Disorders (ASD) and Eating Disorders (ED), as well as the existence of sensory sensitivity alterations in both diagnostic groups.

**Objectives:**

The present study aimed to examine the presence of autistic traits in a sample of adult women diagnosed with different ED, and the concurrent role of autistic traits and sensory sensitivity in both their eating disorder symptomatology and their autism-related eating behaviours.

**Methods:**

Seventy-five women with different ED completed the Eating Attitude Test (EAT-26), the Autism Quotient (AQ), the Ritvo Autism Asperger Diagnostic Scale-Revised (RAADS-R), the Sensory Perception Quotient - Short Form 35 item (SPQ-SF35) and the Swedish Eating Assessment for Autism Spectrum Disorders (SWEAA), which investigates specific eating behaviour related to autism.

**Results:**

12% of the sample scored above the cut-off at both the AQ and the RAADS-R, while 68% scored above the cut-off at the RAADS-R only. We found an association between: i) hypersensitivity in the taste domain and ED severity and autistic-like eating behaviours; ii) hypersensitivity in the vision domain and autistic-like eating behaviours; iii) higher autistic traits and ED severity and autistic-like eating behaviours.

**Conclusions:**

This study confirms the presence of autistic traits in patients with ED and underscores the significance of conducting additional systematical investigations on this topic across all diagnostic categories of ED. It is becoming progressively evident that identifying and measuring the levels of autistic traits in patients with ASD is crucial not only for a better understanding of the causes of these disorders, but also because it would help to tailor specific therapeutic interventions, especially considering the cognitive flexibility issues presented by these patients and the socio-emotional challenges they face. Additionally, this study has laid the foundation for further insights into the relationship between sensory sensitivity and dysfunctional eating behaviours typical of ED and ASD.

**Disclosure of Interest:**

None Declared

